# Psychological, Social and Health-Related Challenges in Spanish Older Adults During the Lockdown of the COVID-19 First Wave

**DOI:** 10.3389/fpsyt.2020.588949

**Published:** 2020-12-03

**Authors:** Raquel Rodríguez-González, David Facal, Alba-Elena Martínez-Santos, Manuel Gandoy-Crego

**Affiliations:** ^1^Department of Psychiatry, Radiology Public Health, Nursing and Medicine, Faculty of Nursing, University of Santiago de Compostela, Santiago de Compostela, Spain; ^2^Health Research Institute of Santiago de Compostela (IDIS), Santiago de Compostela, Spain; ^3^Department of Developmental Psychology, Faculty of Psychology, University of Santiago de Compostela, Santiago de Compostela, Spain; ^4^Department of Pedagogy and Didactics, Faculty of Education Sciences, University of Santiago de Compostela, Santiago de Compostela, Spain

**Keywords:** coronaviral infections, social support, resilience, disaster vulnerability, emotions

## Abstract

**Background:** The global pandemic of COVID-19 has required a population lockdown. Spain has one of the oldest/most aging populations in the world and was one of the most affected countries. We aim to describe the psychological and social implications as well as health-related behaviors as a result of the lockdown in community-dwelling older adults.

**Materials and Methods:** Observational cross-sectional study. A total of 528 participants of over 60 years of age were recruited using snowball sampling technique during the lockdown of the COVID-19 first wave using an anonymous self-administered questionnaire composed of descriptive questions and validated scales for resilience (Brief Resilient Coping Scale) and emotions (Scale of Positive And Negative Experience).

**Results:** Most participants (76.9%) live with other people and have an open space at home (64%). Only 33.7% continued doing activities to promote healthy aging, 65.7% did less physical activity and 25.6% increased their intellectual activity. Most of them (83%) used electronic communication with family and friends to a greater extent, and left the house to run basic errands. Greater scores on resilience showed significant negative correlations with age and negative feelings, and positive correlations with the size of the social network and positive feelings. Lacking an open space at home was associated with more negative feelings.

**Discussion:** Older people are a vulnerable group severely affected by this pandemic crisis at multiple levels, requiring specific interventions to minimize the effects of changes in lifestyle that may be harmful. Detecting needs is essential to improve care and support from community health and social services, both nowadays and in future similar situations.

## Introduction

In January 2020, the World Health Organization (WHO) declared the outbreak of a new Coronavirus disease (COVID-19), to be a public health emergency of international concern. In March, the WHO characterized this disease as a pandemic (1). This led to a situation of global lockdown, where Spain was one of the countries to enforce stricter measures and restrictions. Despite the disease affecting people of all ages, data show that age is a risk factor in terms of complications and associated mortality (2). In this scenario, older people would be an especially vulnerable group to lockdown measures (3). This situation has been particularly important in the case of Spain, which has one of the oldest populations in Europe and is ranked third in the world of countries with the highest life expectancy (4). Several aspects play an important part in personal management of this stressful situation in older adults. These include fear of infection, management of preventive measures, loneliness, support from family and friends, leisure, exercise, and general lifestyle. In this context, the WHO has advised families to provide practical and emotional support by helping seniors to take preventive measures (for example, hand washing), guaranteeing access to their current medications, teaching them simple daily physical exercises to do at home, keeping everyday routines and schedules or helping to create new ones in a new environment, keeping in regular contact with their loved ones (for example, by phone, email, social networks or videoconference) as well as preserving regular sleep routines and healthy foods (1).

This strict and prolonged lockdown situation posed a challenge for the mental health and personal well-being for health and wellness -emotional, cognitive and physical- (5–8). Added to the direct effects of lockdown, the fear of becoming infected with COVID-19 through personal contact must be taken into account. In Spanish citizens confined to their homes, this fear was focused on those tasks that involved leaving home, such as going out to buy food or to the pharmacy. This fear of becoming infected, in interaction with the lockdown scenario, might increase feelings of social isolation and psychological distress, including depressive symptoms and avoidant behaviors (8, 9). To minimize the impact of this situation, personal and social resources become essential. Social support provided through telephone or telematics also become important in lessening the impact. Negative self-perceptions of aging may be related to negative outcomes for older adults and play an important role in the context of lockdown (8, 10). In addition, personal resources such as good management of emotions and resilience will be key to minimize the psychological consequences of this lockdown (11, 12). It is worth noting the importance of resilience, understood as a self-regulating mechanism of protection against the emergence of possible difficult consequences at certain times in life (13, 14), which plays a key role in healthy aging (15). Apart from the potential increases in stress levels and depressive symptoms due to perceived health risk and uncertainty about the future, concerns regarding the effect of the COVID-19 crises in old adults include the curtailing of physical activity and the increases in sedentary behaviors, nutritional changes due to limited shopping and fear of going out, as well as limited access to health services (16).

In addition to the personal and mental health resources of confined people, especially older adults, an important aspect to take into account in the situation of strict lockdown is the housing conditions. Although most people spend the bulk of their lives indoors, buildings are rarely intentionally designed or operated with the goal of promoting mental health (17). Previous works have shown that visual contact with nature has a reliable effect in physiological relaxation (18, 19), but the strict confinements due to the COVID-19 crisis have clearly shown the need to direct outdoor exposure and its potential implication for psychological, social and physical health (20). Amerio et al. (21) studied the relation between mental health and housing conditions, including housing dimension, presence or absence of liveable open space and quality of views, in 8,177 students from a university institute in Milan, Northern Italy, after 3 weeks of strict lockdown. Compared to students with absent to moderate depressive symptoms, students with moderate–severe and severe depressive symptoms lived in smaller apartments, with unusable balconies and poor-quality views from their apartments.

Our objective was to describe the psychological and social implications and health-related behaviors involved in the lockdown due to the COVID-19 pandemic in Galician adults over 60 years old. In order to obtain a more in-depth analysis of the data, groups of participants have been compared, grouping them according to whether they left home and experienced fear when doing so, and according to if they had access to any open space (garden, terrace, balcony) at home. We hypothesize a relation between experiencing fear or discomfort when leaving home, or not leaving home during the lockdown and demographic and social variables related to the risk of COVID-19 and the impact of confinement on the active aging process of the participants. Complementarily, we consider that these demographic and social variables can be predictors of whether old adults leave their home experiencing fear or discomfort, or if they do not leave home during the lockdown.

Regarding the role of psychological variables such as resilient coping and the presence of positive and negative feelings, the current evidence shows that psychological resources were related to the emotional response of the Spanish old adults during the strict lockdown of the COVID-19 first wave (8, 12). We hypothesize a relation between experiencing fear or discomfort when leaving home, or not leaving home during the lockdown and both the psychological resources (resilience) and the psychological response of old adults. Likewise, we speculated if these psychological variables could be predictors of whether old adults leave their home experiencing fear or discomfort, or they do not leave home during the lockdown.

Finally, according to the current evidence about the effect of the exposure to outdoors and its potential implication for psychological and social health, we hypothesize a relation between accesses to any open space (garden, terrace, balcony) at home during the strict lockdown of the COVID-19 first wave and demographic, social and psychological variables.

## Materials and Methods

### Study Design and Data Collection

Observational, cross-sectional study. Non-probability sampling was used using snowball sampling technique. Sample recruitment was initiated through the most representative regional organizations in the promotion of active aging (Galician Association of Permanent Adult Training -ATEGAL-, Program Spaces +60 of Afundación, Red Cross), as well as through the specific university program for older people from our institution. Likewise, participants from the organizations promoting active aging and the university were asked to distribute the questionnaire among their contacts aged 60 and over.

### Instrument

A self-administered questionnaire was distributed electronically (Forms, Microsoft Office 365 available by our university). *Ad-hoc* questions collected information on sociodemographic, psychological, and social data during the COVID-19 lockdown. To evaluate social networks, the specific question: “Approximately, how many close friends or close family do you have? (people with whom you are at ease and can talk about everything you can think of)” from the Spanish version of the Medical Outcomes Study (MOS) questionnaire for social support was used (22, 23). To obtain information regarding resilience, the Spanish version of the Brief Resilient Coping Scale (BRCS) was used (24). It is a 4-item questionnaire to assess optimism, perseverance, creativity and positive growth using a 6-point Likert scale, with higher scores showing greater resilience. To obtain the data on the presence of positive and negative emotions, a part of the Spanish validated version of the Scale of positive and negative experience (SPANE) was used (25). Specifically, two general items (Positive, Negative) and six specific items (Happy, Sad, Afraid, Joyful, Angry, Contented) were chosen. For each one, a number from 1 (Very Rarely) to 5 (Very Often) was selected. Scores were calculated for positive feelings (summation of the positive, happy, joyful, and contented scores) and for negative feelings (summation of the negative, sad, afraid and angry scores).

### Participants

The sample consisted of adults living in the community. Exclusion criteria were (a) being under 60 years old, (b) be living outside Galicia (North-West region of Spain, Europe) during the lockdown declared in March 2020 by the Spanish government due to the COVID-19 pandemic (verified by requiring the postcode), and (c) not providing online informed consent. The study was undertaken between the 20th and 27th April 2020, before the announcement of relief measures which would allow the population to leave home for some hours. A total of 599 participants were recruited, of which 16 were excluded due to being younger than the required age and 55 for not meeting the geographic criteria.

### Ethical Considerations

The study protocol was approved by the Ethics Committee of the University of Santiago de Compostela (reference 040520). Participation was voluntary and all data were processed anonymously according to the current national and European regulations on data protection and patient rights. Participants provided online informed consent.

### Data Analysis

A descriptive analysis was performed by calculating frequencies and percentages for the categorical variables, and means, standard deviations, and association between variables through bivariate correlations for the continuous variables. Specific analyses were performed based on whether participants left the house during lockdown and whether such outings caused discomfort, both questions collapsing into a variable through which three groups were obtained: (a) participants who left and did not feel discomfort, (b) participants who left and felt discomfort, and (c) participants who did not leave the house. Specific analyses were also carried out based on whether the older adults studied had an open space in their home or not. Participants were compared according to both independent variables, through χ^2^ for the categorical dependent variables, and through ANOVAs (and *post-hoc* comparisons with the Bonferroni test) and *t*-test for the quantitative variables.

A hierarchical multinomial logistic regression was complementarily used to identify risk and protective factors of leaving home during the confinement and experiencing fear or not when doing so. Following Losada-Baltar et al. (8), age-related sociodemographic variables connected to risks associated with COVID-19 and how the confinement is experienced (chronological age in years, gender, education level, living alone or not) were included in the first step, followed by personal circumstances during the lock-down associated with such experience and with its impact on the active aging process (participation in activities, physical activity, intellectual activities, diet changes, frequency of calls—video calls made and received), and, finally, psychosocial resources (resilient copying, positive feelings, negative feelings, social network). As has been explained, the available evidence supports that the psychosocial resources and the emotional state play a role in the response of the Spanish old adults during the strict lockdown of the COVID-19 first wave (8, 12).

## Results

### Description of the Participants' Lockdown Situation

The analyzed sample consisted of 528 participants over 60 years old (64.6% women) as shown in Table 1. 76.9% lived with other people at home and 64% had an open space at home (a space belonging to the house, where a person may be outdoors but still at home, for example a garden, terrace or balcony). The vast majority (76.5%) belonged to active aging organizations before lockdown, but only 33.7% continued to carry out active aging activities during this period. Most participants (65.7%) performed less physical activity than before the lockdown, whereas 25.6% of the sample reported an increased intellectual activity. Most participants did not modify their diet during the lockdown and just 15.7% reported eating healthier. Since the lockdown began, 68.9% left their home to run an errand, mainly to go shopping for food or to the pharmacy and mostly wearing mask and gloves (Table 1). Two hundred and thirty people who left their houses (43.6% of the total sample), received information or instructions on the proper use of these protections.

**Table 1:** Sociodemographic, descriptive, psychological, social, and behavioral characteristics of the total sample (*n* = 528) during the COVID-19 lockdown.

**Question**	**Answer**	
How old are you? (years)	69.25 (6.75)	
	***N***	**% of the total sample**
Gender		
Male	187	35.4
Female	341	64.6
What is your educational level?		
Primary	71	13.4
Secondary	77	14.6
Professional training	65	12.3
University studies	315	59.7
Do you live alone?		
Yes	122	23.1
No	406	76.9
Do you have any open spaces (garden, terrace, balcony) at home?		
Yes	338	64
No	190	36
Have you participated in any cultural, recreational or rehabilitation activity for old adults?		
Before lockdown and currently	178	33.7
Before lockdown, but not currently	195	36.9
Neither before lockdown nor currently	155	29.4
During the lockdown, have you done more physical activity than before?		
More	47	8.9
Same	134	25.4
Less	347	65.7
During the lockdown, have you had more intellectual activity than before?		
More	135	25.6
Same	313	59.3
Less	80	15.2
During the lockdown, have you changed your diet?		
Healthier	83	15.7
Same	405	76.7
Less healthy	40	7.6
Have you left home since the beginning of the lockdown?		
Yes	364	68.9
No	164	31.1
Have you felt any kind of discomfort or fear when leaving home?		
Yes	132	25
No	232	43.9
Have you left home wearing a mask?		
Yes	257	48.7
No	107	20.3
Have you left home wearing gloves?		
Yes	259	49.1
No	105	19.9
Has anyone taught you how to use the mask and gloves correctly?		
Yes	230	43.6
No	134	25.4
During the lockdown, have you made more phone calls or video calls to family or friends than before?		
Yes	438	83
No	90	17
During the lockdown, have you received more phone calls or video calls from family or friends than before?		
Yes	439	83.1
No	89	16.9
Do you think you will feel any kind of discomfort when you leave home after the lockdown is over?		
Yes	182	34.5
Indifferent	48	9.1
No	298	56.4
Has this situation given you a more pessimistic view of your immediate future?		
Yes	239	45.3
Indifferent	62	11.7
No	227	43
Do you think your physical health will get worse?		
Yes	88	16.7
Indifferent	90	17.0
No	350	66.3
Do you think your family life will get worse?		
Yes	80	15.2
Indifferent	56	10.6
No	392	74.2
Do you think your social relationships will get worse?		
Yes	168	31.8
Indifferent	42	8
No	318	60.2
Do you think the pandemic will have any beneficial effect?		
Yes	101	19.1
Indifferent	70	13.3
No	357	67.6
**Question/Scale**	**Mean (SD)**	
About how many close friends or close family do you have? (People with whom you feel comfortable and can talk about everything you can think of)	13.59 (12.60)	
Resilient coping (BRCS) score	10.18 (2.76)	
Positive feelings (SPANE-P) score	9.96 (4.65)	
Negative feelings (SPANE-N) score	6.83 (2.70)	

*Results are shown as mean, and standard deviation (SD, in brackets), or frequency and percentage. BRCS, Brief Resilient Coping Scale; SPANE-P, Scale of Positive and Negative Experience—Positive feelings; SPANE-N, Scale of Positive and Negative Experience—Negative feelings*.

The average size of the social network of the participants was 13.59 ± 12.60 people, and 83% made and received more calls from friends or family than before lockdown. Regarding the future, 34.5% think that they will feel some kind of discomfort when leaving home, that their physical health will not worsen (66.3%), nor their family life (74.2%), although this percentage is lower for social relations (60.2%). A large majority believes that this situation will not have a positive effect (67.6%).

BRCS showed significant correlation, in negative direction, with age [*r*_(526)_ = −0.15, *p* = 0.001] and SPANE-N [*r*_(526)_ = −0.37, *p* = 0.001], and positive, with SPANE-P [*r*_(526)_ = 0.34, *p* = 0.001] and with the size of the social network [*r*_(526)_ = −0.14, *p* = 0.001]. SPANE-P also correlated significantly, negatively, with SPANE-N [*r*_(526)_ = −0.21, *p* = 0.001], and positively with the size of the social network [*r*_(526)_ = 0.17, *p* = 0.001].

### Differences Between Participants Who Left Home and Experienced Fear, and Those Who Did Not

Regarding the differences depending on whether the participant had left the house during lockdown, and the impact of these outings (Table 2), significant differences were found for age, gender, educational level, living alone, changes in intellectual activity and diet due to lockdown, use of gloves, pessimistic vision of the future, worsening of physical health and social relationships, and the presence of measured positive and negative feelings, although effect sizes are moderate only for educational level.

**Table 2:** Sociodemographic, descriptive, psychological, social, and behavioral differences depending on whether the participant has left home during lockdown or not.

**Question**	**Leaving home**		**Leaving home**		**Not leaving**		**Test^a^**	***p***	**Effect**
	**without discomfort**		**in discomfort**		**home**				**size^b^**
	**(Group 1**, ***n*** **= 232)**		**(Group 2**, ***n*** **= 132)**		**(Group 3**, ***n*** **= 164)**				
How old are you? (years)	68.56 (5.90)		67.48 (4.91)		71.66 (8.33)		17.24	0.001	0.06
	*N*	%	*N*	%	*N*	%			
Gender									
Male	105	45.3	42	31.8	40	24.4	12.29	0.001	0.19
Female	127	54.7	90	68.2	124	75.6			
What is your educational level?									
Primary	16	6.9	9	6.8	46	28	47.71	0.001	0.21
Secondary	39	16.8	22	16.7	16	9.8			
Professional training	25	10.8	17	12.9	23	14			
University studies	152	65.5	84	63.6	79	48.2			
Do you live alone?									
Yes	68	29.3	31	23.5	23	14	12.65	0.002	0.15
No	164	70.7	101	76.5	141	86			
Do you have any open space (garden, terrace, balcony) at home?									
Yes	144	62.1	83	62.9	111	67.7	1.41	0.493	0.05
No	88	37.9	49	37.1	53	32.3			
Did you participate in any cultural, recreational or rehabilitation activity for old adults?									
Before lockdown, but not currently	64	27.6	40	30.3	51	31.1	1.71	0.789	0.04
Before lockdown and currently	83	35.8	51	38.6	61	37.2			
Neither before lockdown nor currently	85	36.65	41	23.0	52	31.7			
During the lockdown, have you done more physical activity than before?									
More	157	67.7	94	71.2	96	58.5	6.84	0.145	0.08
Same	56	24.1	30	22.7	48	29.3			
Less	19	8.2	8	6.1	20	12.2			
During the lockdown, have you had more intellectual activity than before?									
More	26	11.2	27	20.5	27	16.5	11.85	0.018	0.11
Same	133	57.3	75	56.8	105	64.0			
Less	73	31.5	30	22.7	32	19.5			
Have you changed your diet?									
Healthier	13	5.6	17	12.9	10	6.1	10.71	0.030	0.10
No changes	189	81.5	90	68.2	126	76.8			
Less healthy	30	12.9	25	18.9	28	17.1			
Have you left the house wearing a mask?									
Yes	157	67.7	100	75.8		2.65	0.104	0.09	
No	75	32.3	32	24.2					
Have you left the house wearing gloves?									
Yes	151	65.15	108	81.8		11.48	0.001	0.18	
No	81	34.9	24	18.2					
Has anyone taught you how to use the mask and gloves correctly?									
Yes	146	62.9	84	63.6		0.02	0.893	0.01	
No	86	37.1	48	36.4					
During the lockdown, have you made more phone calls or video calls?									
Yes	194	83.6	108	81.8	136	82.9	0.193	0.908	0.02
No	38	16.4	24	18.2	28	17.1			
During lockdown, have you received more calls/video?									
Yes	191	82.3	110	83.3	138	84.1	0.23	0.891	0.02
No	41	17.7	22	16.7	26	15.9			
Approximately, how many close friends or close family do you have?	13.82 (12.69)	14.03 (13.42)	12.90 (11.84)	0.37	0.692	0.00			
Has this situation given you a more pessimistic view of your immediate future?									
Yes	81	34.9	85	64.4	73	44.5	29.60	0.001	0.17
Indifferent	33	14.2	10	7.6	19	11.6			
No	118	50.9	37	28	71	43.9			
Do you think your physical health will get worse?									
Yes	27	11.6	26	19.7	35	21.3	19.55	0.001	0.14
Indifferent	32	13.8	34	25.8	24	14.6			
No	173	74.6	72	54.5	105	64			
Do you think your family life will get worse?									
Yes	27	11.6	30	22.7	23	14	9.03	0.060	0.09
Indifferent	23	9.9	15	11.4	18	11			
No	182	78.4	87	65.9	123	75			
Do you think your social relationships will get worse?									
Yes	56	24.1	53	40.2	59	36	13.97	0.007	0.12
Indifferent	19	8.2	13	9.8	10	6.1			
No	157	67.7	66	50	95	57.9			
Do you think the pandemic will have any beneficial effect?									
Yes	43	18.5	28	21.2	30	18.3	3.91	0.418	0.06
Indifferent	38	16.4	14	10.6	18	11			
No	151	65.1	90	68.2	116	70.7			
Resilient coping (BRCS)	10.34 (2.62)	9.91 (3.07)	10.17 (2.68)	1.03	0.357	0.00			
Positive feelings (SPANE-P)	10.40 (4.69)	8.74 (4.17)	10.31 (4.83)	6.12	0.002	0.02			
Negative feelings (SPANE-N)	6.35 (2.23)	7.58 (3.11)	6.89 (2.81)	8.98	0.001	0.03			

*Results are shown as mean, and standard deviation (in brackets), or frequency and percentage. BRCS, Brief Resilient Coping Scale; SPANE-P, Scale of Positive and Negative Experience—Positive feelings; SPANE-N, Scale of Positive and Negative Experience—Negative feelings*.

a *F(2, 525), and χ^2^ (2, 528)*.

b $\eta_{p}^{2}$ *with F, Cramer's V with χ^2^*.

Regarding the results of the hierarchical logistic regression for explaining differences in these groups, a good fit of the model was obtained at step 1 when only the sociodemographic variables were included (Chi-square test = 93.99; *p* < 0.01), but a better fit when the personal circumstances during the confinement (Chi-square test = 122.60; *p* < 0.01) and both the personal circumstances and the psychosocial resources (Chi-square test =145.44; *p* < 0.01) were also included. The variables that significantly contributed to the difference between leaving home without feeling discomfort and leaving home with discomfort in the final model were: gender, changes in diet, positive feelings and negative feelings (Table 3). The variables that significantly contributed to the difference between leaving home without feeling discomfort and not leaving home were: age, gender, educative level and not living alone (Table 4).

**Table 3:** Hierarchical logistic regression models comparing the results of those participants leaving home with discomfort with those participants leaving home without discomfort (reference group).

	**B**	**S.E**.	**Wald χ^2^**	***p***	**OR**	**95% CI**
**Model 1**						
Age	−0.03	0.02	1.80	0.24	0.97	0.94–1.01
Gender						
Male	−0.62	0.24	6.89	<0.01	0.54	0.33–0.85
Female	0					
Education level						
Primary	0.04	0.45	0.01	0.94	1.04	0.43–2.52
Secondary	−0.12	0.31	0.15	0.70	0.89	0.49–1.62
Professional training	0.22	0.35	0.40	0.53	1.24	0.63–2.46
University studies	0					
Live alone						
Yes	−0.37	0.26	2.01	0.16	0.70	0.42–1.15
No	0					
**Model 2**						
Age	−0.02	0.02	0.80	0.37	0.98	0.94–1.02
Gender						
Male	−0.71	0.25	7.83	<0.01	0.49	0.30–0.81
Female	0					
Education level						
Primary	−0.05	0.50	0.01	0.91	0.95	0.37–2.42
Secondary	−0.01	0.32	0.00	0.98	0.99	0.53–1.86
Professional training	0.13	0.36	0.13	0.72	1.14	0.56–2.31
University studies	0					
Live alone						
Yes	−0.41	0.27	2.35	0.13	0.66	0.39–1.12
No	0					
Activities for old adults						
Before lockdown and currently	0.37	0.31	1.44	0.23	1.44	0.79–2.63
Before lockdown, but not currently	0.13	0.28	0.23	0.63	1.14	0.66–1.97
Neither before nor currently	0					
Physical activity during lockdown						
More	0.44	0.46	0.89	0.34	1.55	0.62–3.84
Same	0.34	0.50	0.46	0.50	1.40	0.53–3.74
Less	0					
Intellectual activity during lockdown						
More	0.91	0.38	5.80	0.02	2.48	1.18–5.17
Same	0.39	0.28	1.99	0.16	1.48	0.86–2.54
Less	0					
Diet changes						
Healthier	0.15	0.49	0.10	0.75	1.17	0.45–3.03
Same	−0.78	0.32	5.85	<0.05	0.46	0.24–0.86
Less healthy	0					
Have you made more phone calls?						
Yes	−0.13	0.38	0.12	0.73	0.88	0.41–1.85
No	0					
Have you received more phone calls?						
Yes	0.06	0.38	0.03	0.870	1.06	0.51–2.23
No	0					
**Model 3**						
Age	−0.02	0.02	0.67	0.41	0.98	0.94–1.03
Gender						
Male	−0.74	0.26	8.16	<0.01	0.48	0.29–0.79
Female	0					
Education level						
Primary	−0.29	0.50	0.32	0.57	0.75	0.28–2.02
Secondary	−0.05	0.33	0.02	0.88	0.95	0.50–1.82
Professional training	0.16	0.37	0.20	0.65	1.18	0.57–2.43
University studies	0					
Live alone						
Yes	−0.35	0.28	1.57	0.21	0.71	0.41–1.22
No	0					
Activities for old adults						
Before lockdown and currently	0.40	0.31	1.64	0.20	1.50	0.81–2.80
Before lockdown, but not currently	0.12	0.28	0.18	0.67	1.13	0.64–1.97
Neither before nor currently	0					
Physical activity during lockdown						
More	0.40	0.48	0.71	0.40	1.49	0.59–3.81
Same	0.39	0.51	0.58	0.44	1.48	0.54–4.03
Less	0					
Intellectual activity during lockdown						
More	0.72	0.39	3.40	0.65	2.05	0.96–4.40
Same	0.36	0.28	1.66	0.20	1.44	0.83–2.50
Less	0					
Diet						
Healthier	−0.16	0.52	0.10	0.76	0.85	2.37
Same	−0.83	0.33	6.23	<0.05	0.44	0.84
Less healthy	0					
Have you made more phone calls?						
Yes	−0.32	0.40	0.62	0.43	0.73	0.33–1.60
No	0					
Have you received more phone calls?						
Yes	0.20	0.40	0.25	0.61	1.22	0.56–2.65
No	0					
Approximately, how many close friends or close family do you have?	0.01	0.01	0.87	0.35	1.01	0.99–1.03
Resilient coping (BRCS) score	0.05	0.05	0.88	0.35	1.04	0.95–1.16
Positive feelings (SPANE-P) score	−0.08	0.03	7.97	<0.01	0.92	0.87–0.98
Negative feelings (SPANE-N) score	0.14	0.05	8.78	<0.01	1.15	1.05–1.26

*Model 1 includes sociodemographic variables, Model 2 includes sociodemographic variables and personal circumstances during the lockdown, and Model 3 adds psychosocial resources*.

**Table 4:** Hierarchical logistic regression models comparing the results of those participants not leaving home with those participants leaving home without discomfort (reference group).

	**B**	**S.E**.	**Wald χ^2^**	***p*-values**	**OR**	**95% CI**
**Model 1**						
Age	0.05	0.02	8.80	<0.01	1.05	1.02–1.09
Gender						
Male	−1.05	0.25	18.23	<0.01	0.35	0.21–0.57
Female	0					
Education level						
Primary	1.25	0.35	12.77	<0.01	3.51	2.76–6.99
Secondary	−0.27	0.34	0.61	0.44	0.77	0.39–1.50
Professional training	0.61	0.33	3.32	0.07	1.84	0.96–3.56
University studies	0					
Live alone						
Yes	−1.21	0.29	17.07	<0.01	0.30	0.17–0.53
No	0					
**Model 2**						
Age	0.06	0.02	10.49	<0.01	1.06	1.02–1.10
Gender						
Male	−1.10	0.26	18.02	<0.01	0.33	0.20–0.55
Female	0					
Education level						
Primary	1.30	0.38	11.76	<0.01	3.67	1.75–7.73
Secondary	−0.15	0.35	0.17	0.68	0.86	0.43–1.73
Professional training	0.51	0.35	2.15	0.14	1.66	0.84–3.28
University studies	0					
Live alone						
Yes	−1.27	0.30	17.61	<0.001	0.28	0.15–0.51
No	0					
Activities for old adults						
Before lockdown and currently	0.16	0.30	0.29	0.59	1.18	0.65–2.13
Before lockdown, but not currently	0.02	0.28	0.00	0.95	1.02	0.59–1.75
Neither before nor currently	0					
Physical activity during lockdown						
More	−0.67	0.38	3.07	0.08	0.51	0.24–1.08
Same	−0.52	0.41	1.54	0.22	0.59	0.26–1.34
Less	0					
Intellectual activity during lockdown						
More	0.58	0.40	2.14	0.14	1.79	0.82–3.91
Same	0.40	0.28	2.10	0.15	1.49	0.87–2.57
Less	0					
Diet changes						
Healthier	−0.02	0.55	0.00	0.98	0.98	0.34–2.87
Same	−0.63	0.33	3.62	0.06	0.53	0.28–1.02
Less healthy	0					
Have you made more phone calls?						
Yes	0.32	0.39	0.69	0.41	1.38	0.64–2.98
No	0					
Have you received more phone calls?						
Yes	−0.09	0.38	0.06	0.81	0.91	0.44–1.92
No	0					
**Model 3**						
Age	0.06	0.02	10.54	<0.01	1.06	1.02–1.10
Gender						
Male	−1.09	0.26	17.51	<0.001	0.34	0.20–0.56
Female	0					
Education level						
Primary	1.30	0.39	11.34	0.00	3.68	1.72–7.86
Secondary	−0.11	0.36	0.09	0.77	0.90	0.45–1.81
Professional training	0.51	0.35	2.12	0.15	1.65	0.84–3.27
University studies	0					
Live alone						
Yes	−1.25	0.30	17.00	<0.001	0.29	0.16–0.52
No	0					
Activities for old adults						
Before lockdown and currently	0.20	0.31	0.41	0.52	1.22	0.67–2.21
Before lockdown, but not currently	0.03	0.28	0.01	0.92	1.03	0.60–1.77
Neither before nor currently	0					
Physical activity during lockdown						
More	−0.67	0.39	3.01	0.08	0.51	0.24–1.09
Same	−0.52	0.42	1.52	0.21	0.60	0.26–1.36
Less	0					
Intellectual activity during lockdown						
More	0.63	0.40	2.41	0.12	1.87	0.85–4.12
Same	0.42	0.28	2.22	0.14	1.51	0.88–2.61
Less	0					
Diet						
Healthier	0.03	0.56	0.00	0.96	1.03	0.34–3.11
Same	−0.60	0.34	3.19	0.07	0.55	0.29-1.06
Less healthy	0					
Have you made more phone calls?						
Yes	0.27	0.40	0.47	0.50	1.31	0.60–2.86
No	0					
Have you received more phone calls						
Yes	−0.07	0.38	0.03	0.86	0.94	0.44–1.98
No	0					
Approximately, how many close friends or close family do you have?	0.00	0.00	0.08	0.78	1.00	0.98–1.01
Resilient coping (BRCS) score	0.05	0.05	0.81	0.37	1.05	0.95–1.15
Positive feelings (SPANE-P) score	0.00	0.03	0.00	0.98	1.00	0.95–1.05
Negative feelings (SPANE-N) score	0.05	0.05	1.19	0.27	2.06	0.96–1.16

*Model 1 includes sociodemographic variables, Model 2 includes sociodemographic variables and personal circumstances during the lockdown, and Model 3 adds psychosocial resources*.

### Differences According to Availability of Open Spaces at Home

Regarding the differences depending on whether the participant has an open space at home, when comparing those who had an open space with those who did not, no significant differences were found but a tendency toward significant differences was observed in the SPANE-N (with open space: mean = 6.66, S.D. = 2.53; without open space: mean = 7.12, S.D. = 2.96; *t*_2, 525_ = −1.86, *p* = 0.064).

## Discussion

The sample of Galician older adults studied during the lockdown decreed by the Spanish government due to the COVID-19 pandemic is mainly female, with an average age of 69 years and a high educational level. A third of participants have stopped participating in recreational or occupational activities during the lockdown, doing less physical activity. For over 2 months, strict “stay-at-home” or lockdown policies were maintained for all citizens, and houses became the only place where the population could sleep, eat, work, or do exercise. In this scenario, the closure of parks and exercise facilities might have been relevant factors related to the observed decrease of physical activity. Participants mostly increased the use of electronic communication to contact family or friends during the lockdown and considered that they have an important social network that could help them in case of need, stressing the potential positive consequences of the lockdown (26). This result is in line with previous findings showing very good levels of social support in Spanish older adults (27). According to the complex interrelation between social and health factors (28), social and family relations must be considered in tracking psychological changes after the lockdown. As regards the differences between people who left their homes during lockdown, felt fearful or not when doing so, and those who did not leave, most of the effect sizes are small. However, we consider the theoretical interest of these results according to the potential impact of a preventive restrictive quarantine in the social and emotional lives of community-dwelling older adults, and also considering the hierarchical logistic regression supporting these findings.

Our results suggest two broad profiles of older adults experiencing problems resuming daily life after this strongly restrictive quarantine. The logistic regression model shows that leaving home with discomfort was more likely in women who had changed their diet and who experienced fewer positive feelings and more negative feelings. This group might have an increased risk of developing psychopathologies and therefore need more care and attention at the psychosocial level. On the other hand, those who did not leave the house are older, mainly female, with a lower educational level and fewer live alone. Although it has not been measured, it seems reasonable to assume that they present increased limitations of personal autonomy and greater fragility. On a psychological level, this group scored similar to those who left the home and did not feel discomfort and better than those who left the home and presented discomfort. Regarding this profile of old-old women with primary education and not living alone, detecting their health needs is essential to improve care from community health services both now and in similar future situations.

Regarding the role of psychological variables, the lower presence of positive feelings and the higher presence of negative feelings were significant predictors of experiencing fear or discomfort when leaving home. Findings in general population have shown that having a positive affect might help individuals to adopt information-processing strategies during the COVID-19 outbreak that would improve their life satisfaction (29). Regarding resilient coping, BRCS scores were similar to previous studies with Spanish older adults without being in an emergency situation (30). Nevertheless, in our study resilience is not a significative predictor variable, in contrast with results by López et al. (12). More research is needed to clarify to what extent factors like resilience and personal attitudes, including those toward perception of the aging process, play a role in addressing challenges in this pandemic. Likewise, recent studies have investigated the relevance of pre-existing mental health comorbidities in coping with this exceptional situation. Psychiatric patients have shown higher levels of anxiety, depression, insomnia and more health concerns than healthy subjects (31), as well as more frequency of COVID-related stress (32).

In relation to the availability of open spaces at home, Stephens et al. have studied in the last decades the influence of housing conditions on the affective and functional state of older people (33). Studies have shown that housing conditions are even more predictive than the degree of dependency in predicting abandonment of the home and institutionalization (34). In line with Amerio et al. (21), our results point to a greater presence of negative feelings during quarantine in participants who do not have open spaces at home. It has been pointed out that housing design strategies should focus on larger living spaces and visible and accessible green areas (20, 21). These recommendations would be of higher importance in potentially frail older adults. It could be useful, for example, to previously identify vulnerable people lacking open spaces at home to consider rehousing or to give them priority and specific schedules for short outings in case of a new lockdown. An interdisciplinary effort is needed, especially in COVID-19 times, to study the effect of housing conditions on older adults' mental health and to promote healthy living spaces, including professionals from gerontology, mental health, epidemiology, environmental health and urban planning.

The study has several limitations arising from the very early stage of the pandemic when the data were collected, which include the sampling procedure, the exploratory nature of the data collected, and the use of parts of validated scales. The recruitment was carried out looking for a sample as representative as possible through contact with the most representative regional entities in the promotion of active aging. In view of the very urgent nature of data collection, a snowball technique was used as a complementary sampling procedure, as done previously in this exceptional context (9, 12). The resulting sample is greatly female and university-educated, with most participants belonging to active aging organizations and therefore with access to different activities. The wider presence of women is associated with their longer life expectancy as well as greater participation in activities for older adults, and is present in other studies with Spanish population [i.e., Juncos Rabadán et al. (35)]. However, the presence of a higher percentage of older adults with university studies, which could be due to the acquisition of data through an online questionnaire, as well as their belonging to active aging organizations in a specific region of Spain, limit the generalization of the results presented to broader profiles of older adults and other communities. Regarding the cross-sectional nature of the study, available data show how health-related behaviors improved across the lockdown showing how well the Spanish population adapted (36). Longitudinal investigations are needed to determine the emotional impact of the strict quarantine measures adopted by the Spanish government, and its differential impact in community-dwelling old adults. Finally, according to the exploratory nature of the study, we present a high number of group comparisons, which greatly increase the Type I error probability.

In conclusion, early evidence on the effects of lockdown on older adults during the months of March and April 2020 seems to indicate that it is necessary to promote actions to encourage their activity and psychological well-being. Their freedom of movement has been restricted, thus affecting their pattern of active aging (37, 38). In a possible relapse of this situation, certain characteristics such as those described (living alone, presence of psychological distress, type of housing, etc.) cannot easily be directly modified but should be carefully analyzed, since the older population would receive a great deal of support from social and health services. Whereas, other age groups have been encouraged to shift both education and work online to improve health-related behaviors during a possible second wave of the pandemic (36), in community-dwelling old adults the current data points to an importance of psycho-social support. If a strict lockdown is again needed, consideration must be given to improve housing conditions.

## Data Availability Statement

The raw data supporting the conclusions of this article will be made available by the authors, without undue reservation.

## Ethics Statement

The studies involving human participants were reviewed and approved by Ethics Committee of the University of Santiago de Compostela (reference 040520). The patients/participants provided their written informed consent to participate in this study.

## Author Contributions

RR-G and DF: design of the work, acquisition, analysis and interpretation of the data, drafting the work. A-EM-S: design of the work, acquisition and interpretation of the data, revising it critically for important intellectual content. MG-C: conception and design of the work, acquisition and interpretation of the data, drafting the work. All authors contributed to the article and approved the submitted version.

## Conflict of Interest

The authors declare that the research was conducted in the absence of any commercial or financial relationships that could be construed as a potential conflict of interest.
